# Study of genotoxic and cytotoxic effects induced in human fibroblasts by exposure to pulsed and continuous 1.6 GHz radiofrequency

**DOI:** 10.3389/fpubh.2024.1419525

**Published:** 2024-07-31

**Authors:** Luca Massaro, Stefania De Sanctis, Valeria Franchini, Elisa Regalbuto, Gaetano Alfano, Chiara Focaccetti, Monica Benvenuto, Loredana Cifaldi, Antonella Sgura, Francesco Berardinelli, Jessica Marinaccio, Federica Barbato, Erica Rossi, Daniela Nardozi, Laura Masuelli, Roberto Bei, Florigio Lista

**Affiliations:** ^1^Radiobiology Section, Defence Center for Biotechnologies, Defence Institute for Biomedical Sciences, Rome, Italy; ^2^Department of Clinical Sciences and Translational Medicine, University of Rome “Tor Vergata”, Rome, Italy; ^3^Department of Science, University of Rome “Roma Tre”, Rome, Italy; ^4^Department of Experimental Medicine, University of Rome “Sapienza”, Rome, Italy

**Keywords:** radiofrequency, 1.6 GHz, biological effects, protein expression, ultrastructure, EMF, mitotic spindle, genotoxicity

## Abstract

**Background:**

The widespread use of radiofrequency (RF) sources, ranging from household appliances to telecommunications devices and military equipment, raises concerns among people and regulatory agencies about the potential health risks of RF exposure. Consequently, several *in vitro* and *in vivo* studies have been done to investigate the biological effects, in particular non-thermal, of this non-ionizing radiation. To date, this issue is still being debated due to the controversial results that have been reported. Furthermore, the impact of different RF signal modulations on biological systems remains poorly investigated. The present *in vitro* study aims to evaluate the cytotoxicity and genotoxicity of continuous or pulsed 1.6 GHz RF in human dermal fibroblasts (HDF).

**Methods:**

HDF cultures were exposed to continuous and pulsed 1.6 GHz RF, for 2 h, with Specific Absorption Rate (SAR) of 0.4 W/kg. The potential biological effects of 1.6 GHz RF on HDF were assessed with a multi-methodological approach, analyzing the effects on cell cycle, ultrastructure, protein expression, mitotic spindle, CREST stained micronuclei, chromosome segregation and γ-H2AX/53BP1 foci.

**Results:**

1.6 GHz RF exposure modified proteins expression and morphology of HDF. Specifically, the expression of different heat-shock proteins (HSP) (i.e., HSP-90, HSP-60, and HSP-25) and phospho-AKT were affected. In addition, both continuous and pulsed RF modified the cytoskeletal organization in HDF and increased the number of lysosomes, while the formation of autophagosomes was observed only after pulsed RF exposure. Mitotic spindle anomalies were also found after exposure. However, no significant effect was observed on cell cycle, chromosome segregation, CREST-stained micronuclei and γ-H2AX/53BP1 foci.

**Conclusion:**

The results of the present study show the absence of genotoxic damage in 1.6 GHz RF exposed HDF and, although mitotic spindle alterations were observed, they did not have an aneugenic effect. On the other hand, changes in some proteins expression and cell ultrastructure in exposed HDF suggest that RF can potentially induce cell alterations at the morphological and molecular levels.

## 1 Introduction

Natural sources such as solar radiation and geomagnetic fields contribute to electromagnetic radiation background. To date, the widespread use of various instruments and appliances, based on electromagnetic fields (EMFs), significantly increases human exposure to this non-ionizing radiation. Specifically, radiofrequency electromagnetic fields (RF-EMF), including frequencies from 100 kHz to 300 GHz, are mainly used in telecommunication applications such as mobile phones and radio bridges or in medical equipment and even in radars and jammer devices employed in military operations. The increasing exposure to RF-EMF sources raised health concern about their potential effects in humans and especially in people residing in the vicinity of RF sources and in occupationally exposed personnel (1–3).

The most widely accepted mechanism of interaction between RF-EMF and the human body is tissue heating. Based on this well-studied effect, the International Commission on Non-Ionizing Radiation Protection (ICNIRP) and the International Committee on Electromagnetic Safety (IEEE) have established guidelines and exposure limits for protection from adverse health effects of non-ionizing electromagnetic radiation (4, 5).

However, it should be also considered the potential non-thermal biological effects of RF-EMF on cells. Indeed, several studies have reported unclear and conflicting results on this issue. Some possible explanations are the discrepancies in the study design, data collection and reporting (6, 7).

Moreover, the potential different biological effects induced by continuous or otherwise modulated wave signals should be also considered. This issue is of primary importance since different RF wave signals occur in everyday life (2, 8–10). To date, few reports are available on the topic with no consistent evidence (11–13).

Many of the *in vitro* research that investigated the biological effects of RF-EMF focused on DNA damage, chromosome aberrations, enzyme activity, gene/protein expression and cell proliferation.

Most of the studies on RF genotoxicity evaluated DNA damage employing comet assay, to identify both single and double DNA strand breaks (SSBs and DSBs). The majority of these investigations, performed on several cellular models *in vitro* exposed to different RF-EMF frequencies, did not report DNA damage (12, 14–23).

Few studies used a more sensitive method to evaluate the presence of DSBs identifying the phosphorylated H2AX histone (γ-H2AX), alone or in association with p53 binding protein 1 (53BP1). Most of these investigations, performed primarily on human lymphocytes or on human fibroblasts exposed to different frequencies, indicated the absence of DNA damage (13, 24, 25).

The gold standard to assess genotoxicity is the Micronucleus (MN) Test in binucleated cells (BN), which boasts a huge literature (26). MN originates from acentric fragments or chromosome generated by chromosome breaks or chromosome loss, respectively. In both cases, a portion of DNA unable to migrate with the rest of the chromosomes during anaphase will give rise to a MN visible in interphase as a small accessory nucleus beside the main one (27).

Most studies found no significant increase in MN frequency in various cellular models after exposure to different RF-EMF (17, 28, 29). Conversely, MN induction was reported in some *in vivo* studies in which the RF exposures were performed on rat and mice for prolonged periods (30, 31). Interestingly, in some biomonitoring studies in which MN frequency was investigated on buccal mucosa cells of cell phone users, a higher number of MN was observed in heavy mobile phone users respect to light users (32–34).

In order to identify the different MN origin and discriminate between a clastogenic or aneugenic effect, some authors performed CREST analysis using antibodies that recognize inner kinetochore proteins (CENPs). This approach allows to distinguish between centromere negative MN (MN-) indicating chromosome breakage and centromere positive MN (MN+) arising from chromosome loss.

The studies performing MN CREST assay on human fibroblasts *in vitro* exposed to RF-EMF are few and results are conflicting (13, 25).

Genotoxic effects of RF were also assessed analyzing chromosomal aberrations (35). However, the studies mainly performed on human peripheral blood lymphocytes are both limited and controversial (29, 36, 37).

A further area of investigation is the potential impact of RF radiation on the mitotic spindle, a microtubule structure essential for the proper segregation of chromosomes during cell division.

Interestingly, some studies on tubulin reported effects on microtubule formation and polymerization, suggesting that RF may interfere with the mitotic spindle apparatus, leading to impaired cell division and cell death (38, 39).

Many studies evaluating cell cycle modifications were performed to identify potential alterations from normal cell proliferation. Some of these reported activations of a pro apoptotic response (40), accumulation of cells in S phase at 900 MHz (40, 41) and cell senescence at 1.7 MHz RF-EMF (42) in different human cell lines. However, many other studies did not report any effect of RF on cell cycle distribution (13, 20, 25, 43).

In addition to these genotoxic and cytotoxic effects, thermal and oxidative stress was investigated in cells exposed to RF by analyzing the expression of HSP and ROS-related proteins. It has been reported that exposure to 800–950 MHz waves increased the ROS-related phosphorylation of ERK in Rat1 and HeLa cells while it did not affect the expression of HSP-70 in primary thyroid cells (43, 44). Neuron-like cells exposed to 1800 MHz RF-EMF showed decreased HSP-20 expression levels, increased HSP-70 and phosphorylation of HSP-27 expression levels (45).

Furthermore, other studies reported the induction of apoptosis at 1800 MHz EMF with the increase of p53 protein levels and the activation of caspase 3 in mouse and human *in vitro* models (46), while the activation of a pro-inflammatory responses was observed in microglial cells and astrocytes after exposure to 1800 MHz (47).

Very few studies analyzed the effects of RF focusing on subcellular components by ultrastructural analysis. No structural changes were reported in a study performed on human glioblastoma cell lines exposed to 1950 MHz RF-EMF (48), while structural alterations with mitochondrial damage were observed in normal rat astrocytes after 48 h exposed at the same frequency (49). Activation of autophagy, showed by the formation of autophagosomes, was observed in mouse spermatocyte-derived cells (GC-2) and in spiral ganglion neurons (SGN) exposed to 1800 MHz waves (GSM talk signal) (50, 51).

In this unclear scientific context, the objective of this study is to provide a better understanding of the potential non-thermal biological effects of RF at the cellular level. For this purpose, HDF were exposed to 1.6 GHz RF with both continuous wave (CW) and pulsed wave (PW) signals at a SAR value of 0.4 W/kg for 2 h. In order to obtain a more comprehensive result, the genotoxic and cellular effects were evaluated employing several endpoints: cell cycle, γ-H2AX/53BP1 foci, CREST-stained MN, α- and γ-Tubulin of mitotic spindle, chromosome segregation, protein expression and cellular ultrastructure.

## 2 Materials and methods

### 2.1 Cells

Human dermal fibroblast (HDF) (Cell Applications, Inc., San Diego, CA, United States) primary cells, derived from normal human dermis, were maintained in Dulbecco’s Modified Eagle Medium (DMEM) (Euroclone, Pero, Italy), supplemented with 10% foetal bovine serum (Euroclone), 1% 200 mM L-glutamine, 1% penicillin/streptomycin (Thermo Fisher Scientific, Monza, Italy) and 1% non-essential amino acids (Euroclone) and were grown at 37°C in an incubator with an atmosphere of 5% CO_2_. In order to prevent problem related to cell senescence/proliferation and to ensure the reproducibility of the results, all the experiments were performed on primary fibroblasts at the same passage (number 6).

Cells were then seeded at a density of 1×10^5^ in 35 mm diameter polystyrene Petri dishes 24 h (Cell cycle analysis, Ultrastructural analysis, and γ-H2AX/53BP1 foci analysis) or 48 h (Analysis of mitotic spindle perturbations/anomalies, Cytokinesis-block Micronucleus assay and CREST staining, Chromosomesegregation analysis) before exposure and maintained in complete medium throughout the exposure (2 h) and during post-exposure incubation. In order to maintain cells under appropriate conditions (37°C, 5% CO_2_), all exposures sessions were performed in the incubator.

### 2.2 Exposure system and dosimetry

To expose the human fibroblasts, an exposure system based on a TEM-cell has been used. The core of the exposure system is the RF structure working in the frequency range 800 MHz – 3 GHz that is placed inside the incubator. The Petri dishes were located inside it. The RF structure of the exposure system is a Transverse Electromagnetic Mode (TEM) cell that allows exposing each Petri dish to the same E-field. The TEM permitted to allocate at the same time four 35 mm Petri dishes.

The chain to generate the appropriate electric field is based on four blocks: the RF signal generator Keysight N9310A (Keysight Technologies) generates the RF signal that is amplified by the FLG-10CA (Frankonia Group) power amplifier to feed the open TEM cell TEM3000 (Montena), placed into the “Galaxy S CO_2_ Incubator” (Model n°170–200) in order to maintain a stable environment and optimal thermodynamic conditions; moreover, its inner-walls were covered with a layer of radio-absorbing panels in order to prevent the metal walls inside the incubator from creating unwanted reflections. The EM exposure conditions were constantly monitored by the presence of two power meters connected to a bidirectional coupler to control the incident power and the reflected power. Finally, the whole system also included a PC for the continuous control of the power levels.

The biological samples were exposed for 2 h to CW or PW, at 1.6 GHz RF-EMF. The PW is modulated in amplitude with a pulse period of 2 ms and a duty cycle of 27.5%.

The chosen dose to be delivered to the biological samples was a SAR equal to 0.4 W/kg. The uniformity of the exposure and the dose to the samples were calculated by means of numerical simulations, revealing an exposure homogeneity of about 70%.

In the end, the temperature was also monitored through a fiber-optic temperature probe (FISO Fiber Optic Temperature) connected to a designated temperature read-out (FOR-1 Single Channel Portable Readout) inserted horizontally in the biological samples. The measurements revealed that the maximum temperature increase during 2 h of exposure was 0.35°C for both CW and PW exposure. More details about the exposure system and its characterization are included in the Supplementary Material. Each session included three or four Petri dishes exposed to RF placed inside the TEM cell shielded by radio-absorbing panels and three or four non exposed (sham) Petri dishes placed in the same incubator. At least three experimental replicates were performed for each endpoint evaluated.

### 2.3 Cell cycle analysis

Analysis of cells was performed 2 and 24 h after the end of exposure. Adherent and suspended cells were harvested, centrifuged at 300 g for 10 min and washed twice with cold phosphate buffered saline (PBS, Euroclone, Pero, Italy).

Cell pellets were resuspended in 70% ethanol and incubated 1 h at −20°C. Cells were then washed twice with cold PBS, centrifuged at 300 g for 10 min, incubated for 1 h in the dark with propidium iodide (PI, 25 μg/mL final concentration in 0.1% citrate and 0.1% Triton X-100) and analyzed with flow cytometry using a FACSCalibur cytometer running CellQuest Pro 5.2 software (BD Biosciences, San Jose, CA, United States) A total of 20,000 events were collected (52).

### 2.4 Ultrastructural analysis

For ultrastructural analysis, 2×10^5^ cells were plated in 35 mm Petri dishes 24 h before exposure and allowed to adhere in incubator. For each experimental condition, four 35 mm Petri dishes were used for a total of about 8×10^5^ cells to be processed, and experiments were performed in triplicate. Analysis of cells was performed 2 and 24 h after the end of exposure. Cells were fixed in 2.5% glutaraldehyde in PBS pH 7.4 at 4°C and then observed and photographed by inverted optical microscope (53, 54). After 24 h, fixed cells were collected, post-fixed with 1.33% osmium tetroxide, dehydrated in graded alcohols, and then embedded in Epon 812 resin (Fisher Chemical Co., Dallas, TX, United States). The resin was allowed to polymerize in a dry oven at 60°C overnight, and specimens were cut on a Reichert ultra-microtome (Leica Microsystems GmbH, Wetzlar, Germany). Three ultrathin sections were mounted on copper grids for each experimental condition for each experiment, stained with UranyLess staining (Electron Microscopy Sciences, Hatfield, PA, United States) and lead citrate, and observed under a Philips Morgagni 268D transmission electron microscope (Thermo Fisher Scientific, Waltham, MA, United States) (55).

### 2.5 Western blotting

Analysis of cells was performed 2 and 24 h after the end of exposure. Cells were harvested, washed twice with cold PBS, and lysed in RIPA lysis buffer (Triton X-100 1%, SDS 0.1%, NaCl 200 mM, Tris–HCl 50 mM pH 7.5, PMSF 1 mM, Na_3_VO_4_ 1 mM). After 30 min at 4°C, the mixtures were centrifuged at 12,000 g for 15 min and the supernatants were analyzed by Western blotting. For immunoblotting analysis, 30–50 μg of cell lysates were resolved in 10–12% SDS-PAGE and then transferred to nitrocellulose membranes. Equal loading of protein was verified by Ponceau staining of the membranes and by analysis of actin expression. After blocking, the membranes were incubated with specific primary antibodies overnight at 4°C. Mouse anti-human HSP-90 alpha antibody (cat. no. SMC-108C; 1:1000), mouse anti-human HSP-70 antibody (cat. no. SMC-100; 1:2000), mouse anti-human HSP-60 antibody (cat. no. SMC-110; 1:20000), mouse anti-human HSP-25/27 antibody (cat. no. SMC-114; 1:5000) were obtained from StressMarq Biosciences Inc. (Victoria, BC, United States). Mouse anti-human ERK antibody (cat. no. 610030; 1:200) and mouse anti-human phospho-ERK1/2 (pT202/pY204) antibody (cat. no. 612358; 1:200) were obtained from BD Pharmingen (BD Biosciences, San Jose, CA, United States). Rabbit anti-human Akt antibody (cat. no. #4691S; 1:1000) and rabbit anti-human phospho-Akt (S473) antibody (cat. no. #4060S; 1:500) were obtained from Cell Signaling Technology (Danvers, MA, United States). Rabbit anti-actin (cat. no. A5060; 1:500) was obtained from Merck-Italy-Sigma Aldrich (St. Louis, MO, United States). After washing, the filters were incubated with goat anti-mouse (cat. no. A4416; 1:5000; Merck-Italy-Sigma Aldrich) or -rabbit IgG (cat. no. A6154; 1:10000; Merck-Italy Sigma Aldrich) peroxidase conjugated secondary antibodies and developed by chemiluminescence as previously described (56). The expression of several heat shock proteins (HSPs), ERK, phospho-ERK, AKT and phospho-AKT has been evaluated. A densitometric analysis of autoradiographic bands was performed with Image J software (National Institutes of Health, United States) after blot scanning (56, 57). For each time point analyzed, the comparison of the intensity of immunoreactive bands was evaluated between sham sample and the relative exposed sample.

### 2.6 Analysis of mitotic spindle perturbations/anomalies by α- and γ-tubulin immunostaining

Immediately after exposure, cells were fixed in ice-cold methanol for 10 min and air-dried. Afterward, samples were blocked using 5% bovine serum albumin (Sigma Aldrich, United States) for 1 hour at room temperature (RT). Later cells were incubated with mouse monoclonal antibody for α-tubulin (Sigma Aldrich, United States) and rabbit polyclonal antibody for γ-tubulin (Sigma Aldrich, United States) in 5% BSA overnight at 4°C in wet chamber. Cells were incubated for 1 h at 37°C in anti-mouse Alexa Fluor 546-conjugated antibody (Thermo Fisher Scientific, Life technologies, United States) and anti-rabbit Alexa Fluor 488-conjugated antibody (Thermo Fisher Scientific, Life technologies, United States). Cells were counterstained with DAPI (4,6-diamidino-2 phenylindole, Sigma Aldrich, United States) in Vectashield (Vector Laboratories, United States). Images were captured at a 63X magnification with an AxioImager Z2 (Zeiss, Jena, Germany) equipped with a charge-coupled device (CCD) camera. Two hundred mitoses per sample were counted and spindle anomalies were classified according to Baudoin and Cimini (58).

### 2.7 Cytokinesis-block micronucleus assay and CREST staining

Cytokinesis-blocked binucleated (BN) cells were obtained treating for 24 h exposed and sham samples with Cytochalasin-B (3 mgmL−1 final, Sigma-Aldrich) in order to block cytokinesis. In addition, a modified version of the CBMN assay protocol was also used to focus the analysis mainly on cells that were in M-phase during RF exposure. In this protocol Cytochalasin-B was added 2 h prior the exposure and cells were harvested and fixed 4 h after the exposure. Slides were then processed for anti-kinetochore staining as previously described (59). Briefly, CREST anti-kinetochore antibody (Antibody Inc., Davis CA) and FITC-conjugated secondary antibody (Sigma Immunochemicals, St. Louis) were used. Cells were counterstained with DAPI in Vectashield and analyzed by fluorescence microscopy using an AxioImager Z2 microscope equipped with a CCD camera controlled by the ISIS software (MetaSystems, Altlussheim, Germany). MN frequencies were assessed counting at least 1,500 BN cells for each experiment. In agreement with the internationally accepted criteria (27), only MN that were not larger than one-third of the diameter of the main nucleus, did not overlap the main nucleus, and had distinct borders were included in the scoring. MN were classified for the presence (CREST-positive MN) or absence (CREST-negative MN) of kinetochore fluorescent signals. As positive control for CREST-negative MN and CREST-positive MN, cells were either irradiated using 1Gy X-rays or treated with 10 ng/mL colchicine.

### 2.8 Chromosome segregation analysis

For the CBMN assay, standard and modified protocols were used to assess non-disjunction events. Immediately after exposure methanol and acetic acid (5:1) fixed cells were dropped onto slides, airdried and immediately utilized for FISH staining. Briefly, samples and centromeric probes (chr 4, 10 and 17) (Metasystems, Germany) were denatured simultaneously by heating on a hotplate at 75°C for 2 min and hybridization was performed overnight at 37°C in a humidified chamber. Slides were washed in 2X SSC/0.05% Tween 20 (pH 7.0) for 30 s at RT and 0.4X SSC for 4 min at 74°C, and then counterstained with DAPI in Vectashield. Samples were observed using fluorescent microscope AxioImager M1 (Carl Zeiss, Germany) equipped with a CCD camera. At least 1,000 BN cells were analyzed. To restrict the scoring to the first mitosis after exposure and to exclude technical artefacts, only binucleated cells with the correct number of hybridization signals were analyzed (60). We considered normal BN, cells with a chromosomic pattern of 2 + 2 (2 signals for each chromosome/fluorochrome on each daughter cells). Binucleated cells with an unequal chromosome distribution of one of the fluorochromes, as 3 + 1 or 4 + 0, were considered aneuploid.

### 2.9 γ-H2AX/53BP1 foci analysis

Analysis of γ-H2AX/53BP1 foci was performed 30 min, 2 and 24 h after the end of cell exposure. At the different timepoints after exposure, cells were fixed using 2% formaldehyde/PBS for 5 min, permeabilized using 0.5% Triton-X/PBS for 5 min, and blocked using 1% bovine serum albumin (BSA) (Sigma-Aldrich, Italy)/PBS for 10 min. Cells were then incubated with a combination of 1:500 mouse monoclonal anti-γ-H2AX antibody (Merk Millipore Sigma-Aldrich,) and 1:1000 rabbit polyclonal anti-53BP1 antibody (Calbiochem, Sigma-Aldrich) in 1% BSA/PBS for 45 min at room temperature (RT) in a wet chamber. Subsequently, cells were washed in 1% BSA/PBS three times for 3 min and incubated in 1:500 anti-mouse Alexa Fluor 488 conjugated antibody (Molecular Probes, Life technologies, ThermoFisher Scientific) and 1:500 anti-rabbit Alexa Fluor 555 Goat anti Rabbit IgG (Molecular Probes, Life technologies, ThermoFisher Scientific) for 30 min at RT in a wet chamber in the dark. The cells were extensively washed with PBS, dried, and mounted with DAPI in Vectashield solution. Coverslips with cells were turned upside down on the slide and the edges were sealed using nail polish. Slides were analyzed with an epifluorescence microscope (Imager Z1, Carl Zeiss, Germany) equipped with a CCD camera. The automated image acquisition was performed using Metafer 4 software (version 3.6.9, from MetaSystems, Germany). The first step of the automated scanning of the slides was performed at 10X magnification for nuclei detection (13). Subsequently, the nuclei were scored with a 63X objective to detect the green and red fluorescence signals identifying, respectively, γ-H2AX and p53BP1 foci.

For each time point evaluated, one slide was analyzed. The colocalized γ-H2AX/53BP1 were quantified in a maximum of 250 cells using Metafer4 software and a costume-made evaluation algorithm (classifier).

As positive control for γ-H2AX/53BP1 colocalization foci analysis, cells were irradiated using 1 Gy γ-rays.

### 2.10 Statistical analysis

The statistical analyses were performed using different tests according to the assay. Data sets of CREST-stained MN were analyzed by a two way-ANOVA followed by Dunnett test. A Chi-squared test was employed to analyze data sets of non-disjunction analysis. Data sets from cell cycle analysis tubulin analysis, and differences in the intensity of immunoreactive bands were evaluated by two-tailed unpaired Student’s *t*-test, while γ-H2AX/53BP1 colocalization foci analysis was evaluated by a two-tailed paired Student’s *t*-test. The threshold for statistical significance was set at *p*-value <0.05.

## 3 Results

### 3.1 Cell cycle analysis

To investigate the cell cycle distribution of sham and exposed primary HDF, FACS analysis of DNA content has been performed. Results obtained from the HDF exposed to both type of signals were compared to those of the corresponding sham samples. No significant variations in the different phases of the cell cycle were observed 2 and 24 h after HDF-CW or -PW exposure (Table 1).

**Table 1:** FACS analysis.

1.6 GHz	Time point after exposure	Sample	Sub-G1*		G0/G1		S		G2/M	
			Mean ± SD	*p* ^a^	Mean ± SD	*p*	Mean ± SD	*p*	Mean ± SD	*p*
CW	2 h	Sham	1.71 ± 0.49		67.96 ± 1.22		4.66 ± 0.52		25.95 ± 0.99	
		Exposed	1.91 ± 0.18	NS	66.88 ± 2.15	NS	4.67 ± 0.50	NS	26.81 ± 1.58	NS
	24 h	Sham	2.61 ± 0.40		78.19 ± 1.78		5.02 ± 0.30		14.43 ± 2.05	
		Exposed	2.33 ± 0.18	NS	80.36 ± 0.71	NS	4.77 ± 0.52	NS	12.80 ± 1.39	NS
PW	2 h	Sham	1.67 ± 1.27		72.06 ± 6.85		4.88 ± 1.03		21.23 ± 6.77	
		Exposed	1.36 ± 0.49	NS	71.31 ± 5.74	NS	5.33 ± 1.23	NS	22.27 ± 5.30	NS
	24 h	Sham	0.83 ± 0.21		82.69 ± 2.24		3.55 ± 0.52		13.14 ± 2.24	
		Exposed	0.69 ± 0.02	NS	80.85 ± 0.61	NS	3.75 ± 0.08	NS	14.85 ± 0.75	NS

Percentages of HDF in the different phases of the cell cycle after exposure to 1.6 GHz CW or PW RF are reported. *Percentage of cells in subG1, G0/G1, S, and G2/M phases was calculated with CellQuest Pro 5.2 software. The results reported are mean values ± standard deviation (SD) from three independent experiments. NS, not significant.

a Significance of the effects comparing exposed vs. sham HDF cells was calculated employing the 2-tailed unpaired Student’s *t*-Test.

### 3.2 Morphological analysis

**Figure 1:**
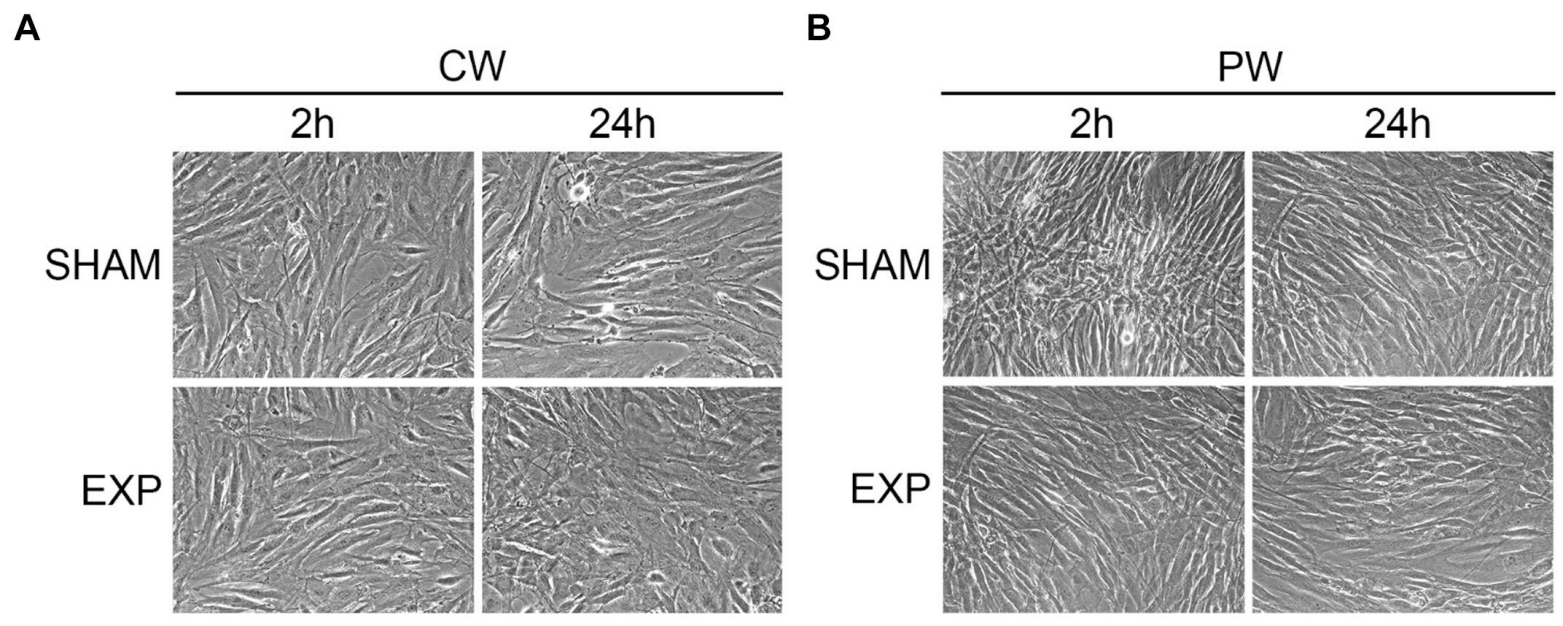
Morphological analysis of HDF after exposure using inverted microscope. Analysis of exposed HDF 2 and 24 h after exposure to 1.6 GHz-CW **(A)** or PW **(B)**. Original magnification x200.

To investigate potential morphological differences induced by different RF exposures, sham and exposed HDF were processed for ultrastructural analysis. Firstly, cells were fixed two and twenty-four hours after exposure and observed by inverted microscope. No morphological differences were found between sham and cells exposed to CW or PW in the nuclei/cytoplasm ratio, cellular shape, presence of vacuoles in the cytoplasm or cellular morphology (Figure 1).

For ultrastructural analysis, 10 to 15 cells per experimental condition were analyzed in triplicate. All the reported morphological modifications were observed in at least 50% of the exposed cells.

**Figure 2:**
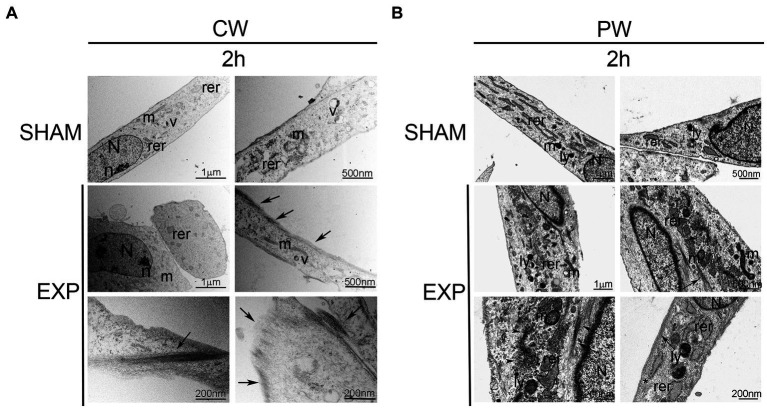
Ultrastructural analysis of HDF 2 h after exposure to 1.6 GHz CW or PW RF. **(A)** HDF observed 2 h after CW exposure. **(B)** HDF observed 2 h after PW exposure. SHAM, control cells; EXP, exposed cell; m, mitochondria; rer, rough endoplasmic reticulum; v, vacuoles; ly, lysosomes; N, nucleus, n, nucleolus; arrows, intermediate filaments; arrowheads, microvesicles; *, pinocytic vesicles. Bars correspond to 1 μm, 500 nm and 200 nm as indicated.

**Figure 3:**
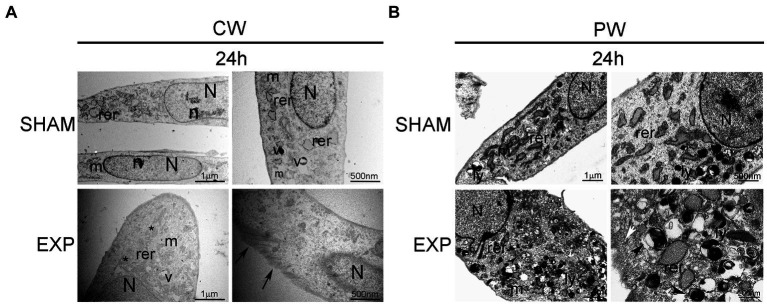
Ultrastructural analysis of HDF 24 h after exposure to 1.6 GHz CW or PW RF. **(A)** HDF observed 24 h after CW exposure. **(B)** HDF observed 24 h after PW exposure. SHAM, control cells; EXP, exposed cell; m, mitochondria; rer, rough endoplasmic reticulum; v, vacuoles; ly, lysosomes; N, nucleus; n, nucleolus; *, fragmentation of RER; arrows, intermediate filaments; arrowheads, autophagic vacuole; white arrow, pinocytic vesicles. Bars correspond to 1 μm, 500 nm and 200 nm as indicated.

Ultrastructural analysis performed on not exposed HDF showed elongated cells with elongated centrally located nuclei, essentially formed by euchromatin with poor heterochromatin and well-organized nucleoli. Condensed or dilated mitochondria, dilated rough endoplasmic reticulum, few vacuoles and lysosomes were occasionally observed (Figures 2, 3).

Cells exposed to 1.6 GHz CW or PW RF, analyzed 2 h after exposure, showed an elongated morphology with centrally located elongated nuclei. No differences in the organization of subcellular organelles were visible in cells 2 h after CW exposure compared to control cells, except for a substantial increase in the polymerization of thin and intermediate filaments below the plasma membrane and in the cytoplasm (Figure 2A). A slight increase in lysosomes and a dilatation of rough endoplasmic reticulum along with an increase of the polymerization of thin and intermediate filaments were visible below the plasma membrane and in the cytoplasm of exposed cells 2 h after PW RF exposure compared to control cells. Indeed, microvesicles, probably representing the fragmentation of the rough endoplasmic reticulum, and pinocytic vesicles were visible in the cytoplasm (Figure 2B).

An increase in the polymerization of cytoskeletal filaments was observed 24 h after exposure to 1.6 GHz RF CW. In addition, CW-exposed HDF appeared larger than the sham cells 24 h after exposure, and they showed a rough endoplasmic reticulum fragmentation in the cytoplasm (Figure 3A). A substantial increase in lysosomes was observed in the cytoplasm of HDF 24 h after exposure to 1.6 GHz PW RF (Figure 3B); several lysosomes were double membrane-surrounded, thus suggesting the formation of autophagosomes and activation of autophagy as pro-survival cell signaling. Dilatation and fragmentation in microvesicles of the rough endoplasmic reticulum along with pinocytic vesicles were observed in the cytoplasm (Figure 3B).

### 3.3 Protein expression

**Figure 4:**
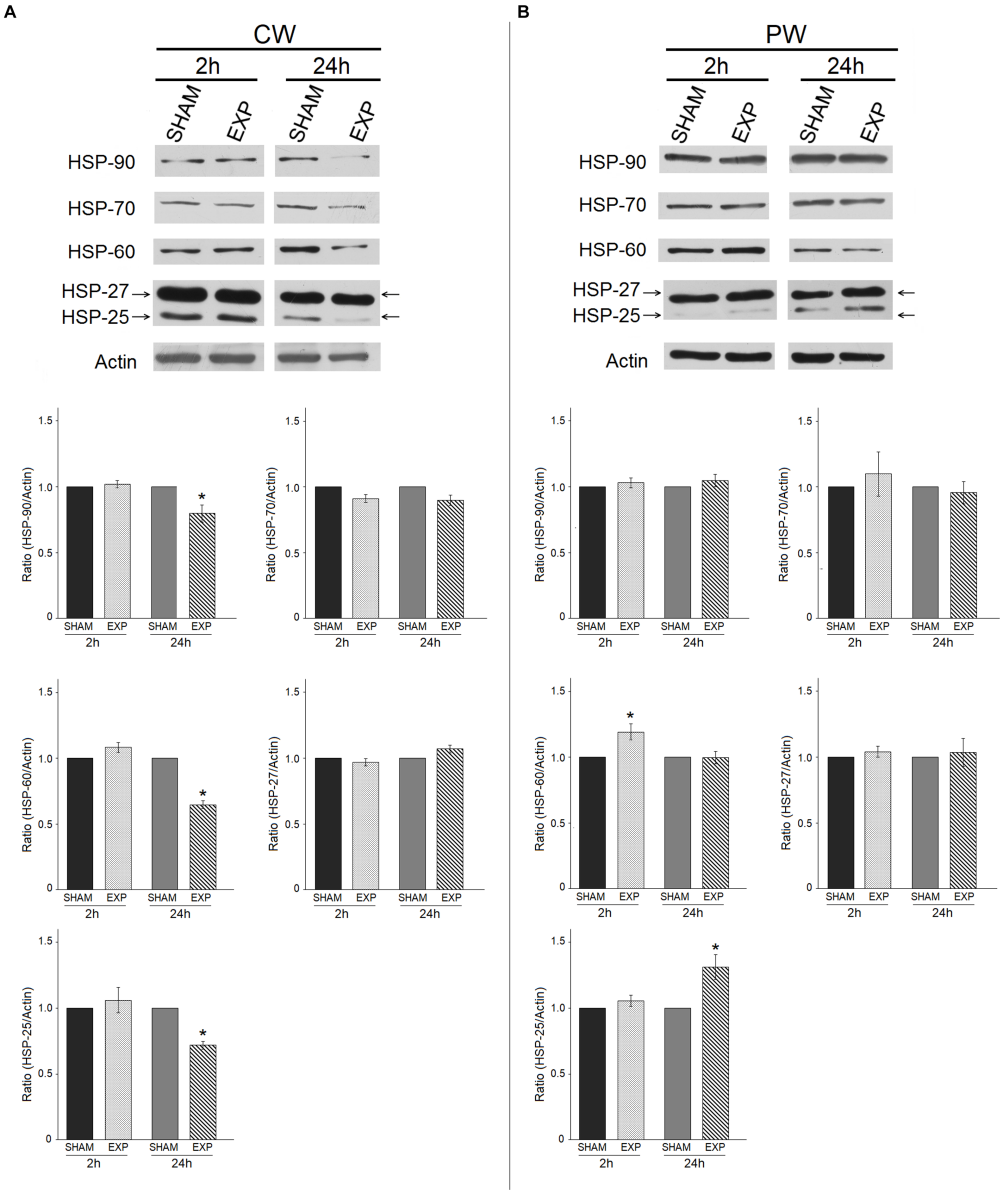
Effect of 1.6 GHz CW or PW RF on heat shock proteins (HSP-90, −70, −60, −25/27). Western blotting was performed on sham cells and exposed cells 2 h and 24 h after exposure to 1.6 GHz CW **(A)** or PW **(B)** RF. Actin was used as an internal control. Densitometric ratios and statistical analysis are reported. Data are expressed as the mean ± SD of two independent experiments (*p ≤ 0.05).

Effects on protein expression following RF-EMF exposure on HDF were evaluated by western blotting. In particular, we analyzed the expression of HSPs, which might vary their expression or activation in stress conditions (e.g., exposure to RF). The modulation of HSPs protein expression after RF exposure was evaluated on HDF 2 h and 24 h after the exposure at 1.6 GHz CW or PW RF. RF-EMF exposure did not significantly alter the expression of HSPs 2 h after CW RF exposure, while decreased the expression of HSP-90 (*p* ≤ 0.05), HSP-60 (*p* ≤ 0.05), HSP-25 (*p* ≤ 0.05) in HDF cells 24 h after the CW RF exposure (Figure 4A). PW radiation did not significantly affect the expression of HSP-90, −70 and − 27 in HDF, 2 and 24 h after the RF exposure (Figure 4B). Conversely, 1.6 GHz PW RF exposure increased the expression of HSP-60 (*p* ≤ 0.05), and HSP-25 (*p* ≤ 0.05) 2 h and 24 h after PW RF exposure, respectively (Figure 4B). We performed a pilot experiment with other two different sham samples placed in the incubator simultaneously (SHAM OFF: Petri dishes with HDF placed in the incubator when the RF-EMF is switched OFF; SHAM TEM OFF: Petri dishes with HDF placed in the TEM cell when the RF-EMF is switched OFF) in addition to our SHAM and exposed (EXP) samples. We decided to perform this pilot experiment 24 h after CW exposure, because we observed most of protein expression differences at this time point. No difference between the three SHAM samples was found. On the other hand, we observed a decrease of HSP-90, −60, −25 in the exposed sample as compared to all the three SHAM samples, thus confirming our previous results (Supplementary Figure S4).

We also investigated the effect of RF exposure on the expression and phosphorylation of ERK1/ERK2 (ERK1/2) and AKT proteins, which play a key role in many physiological processes and control various responses within the cell, depending on cell type and stimuli, such as differentiation, cell cycle, transcription, translation, metabolism, autophagy (61, 62). The levels of phosphorylated ERK1 and ERK2 (pERK1/2), and AKT (pAKT) proteins were compared with the total ERK and AKT protein levels, respectively.

**Figure 5:**
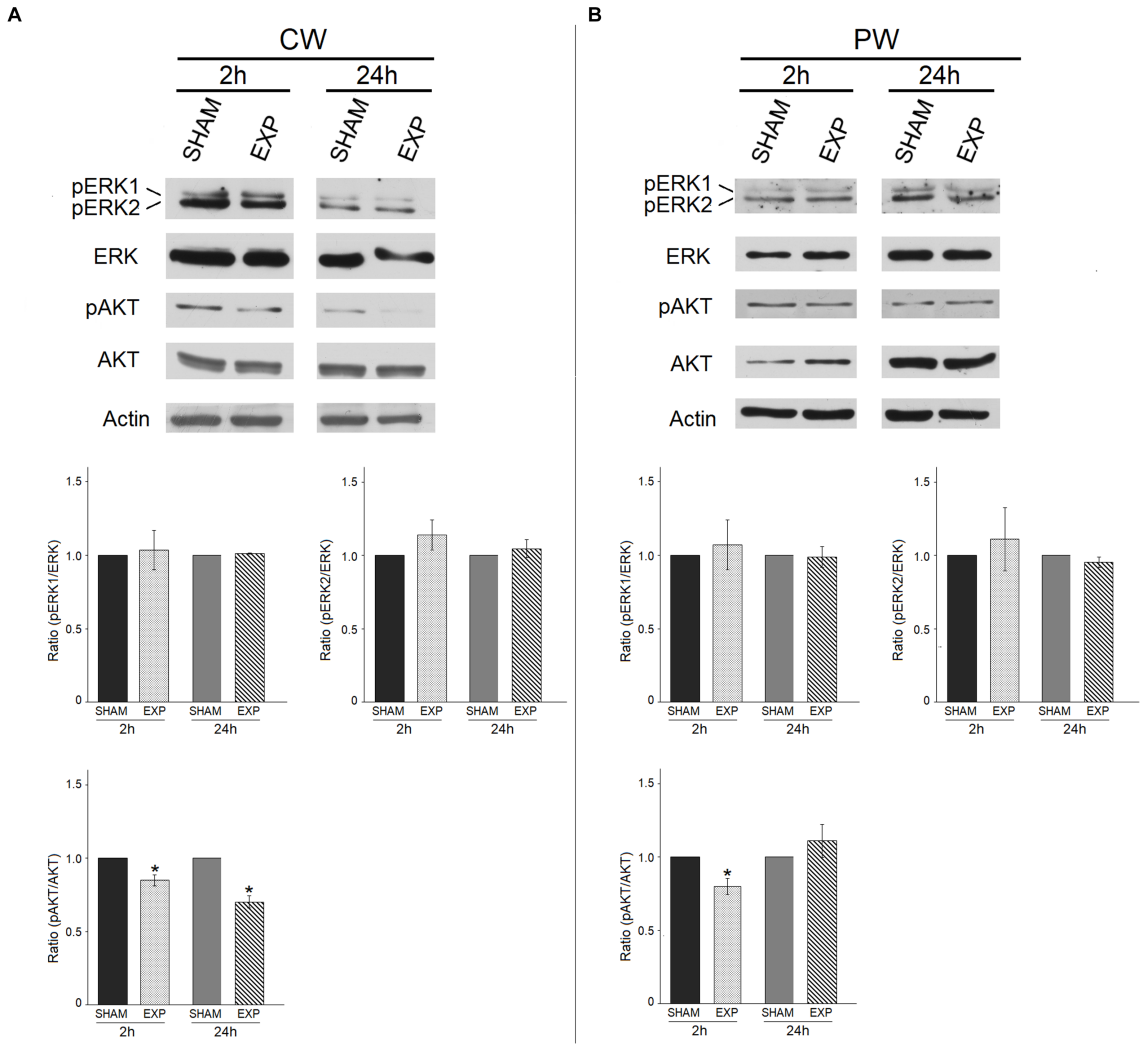
Effect of 1.6 GHz CW or PW RF on pro- survival signaling proteins (ERK and phospho-ERK, AKT and phospho-AKT). ERK1/ERK2 and AKT phosphorylation status in sham cells and exposed cells (EXP) 2 h and 24 h after exposure to 1.6 GHz CW **(A)** or PW **(B)** RF. The levels of phosphorylated ERK1/ERK2 and AKT were compared with those of the total ERK and AKT proteins, respectively. Actin was used as an internal control. Densitometric ratios and statistical analysis are reported. Data are expressed as the mean ± SD of two independent experiments (*p ≤ 0.05).

CW or PW RF exposures did not significantly alter the basal or phosphorylated ERK1/2 protein expression levels in HDF 2 or 24 h after exposure (Figures 5A,B). Conversely, CW and -PW RF exposures significantly decreased activated phosphorylated pro-survival kinase AKT (pAKT) protein levels compared to sham HDF 2 h and 24 h after CW exposure and at 2 h after PW exposure (*p* ≤ 0.05) (Figures 5A,B).

### 3.4 Analysis of mitotic spindle perturbations/anomalies

**Figure 6:**
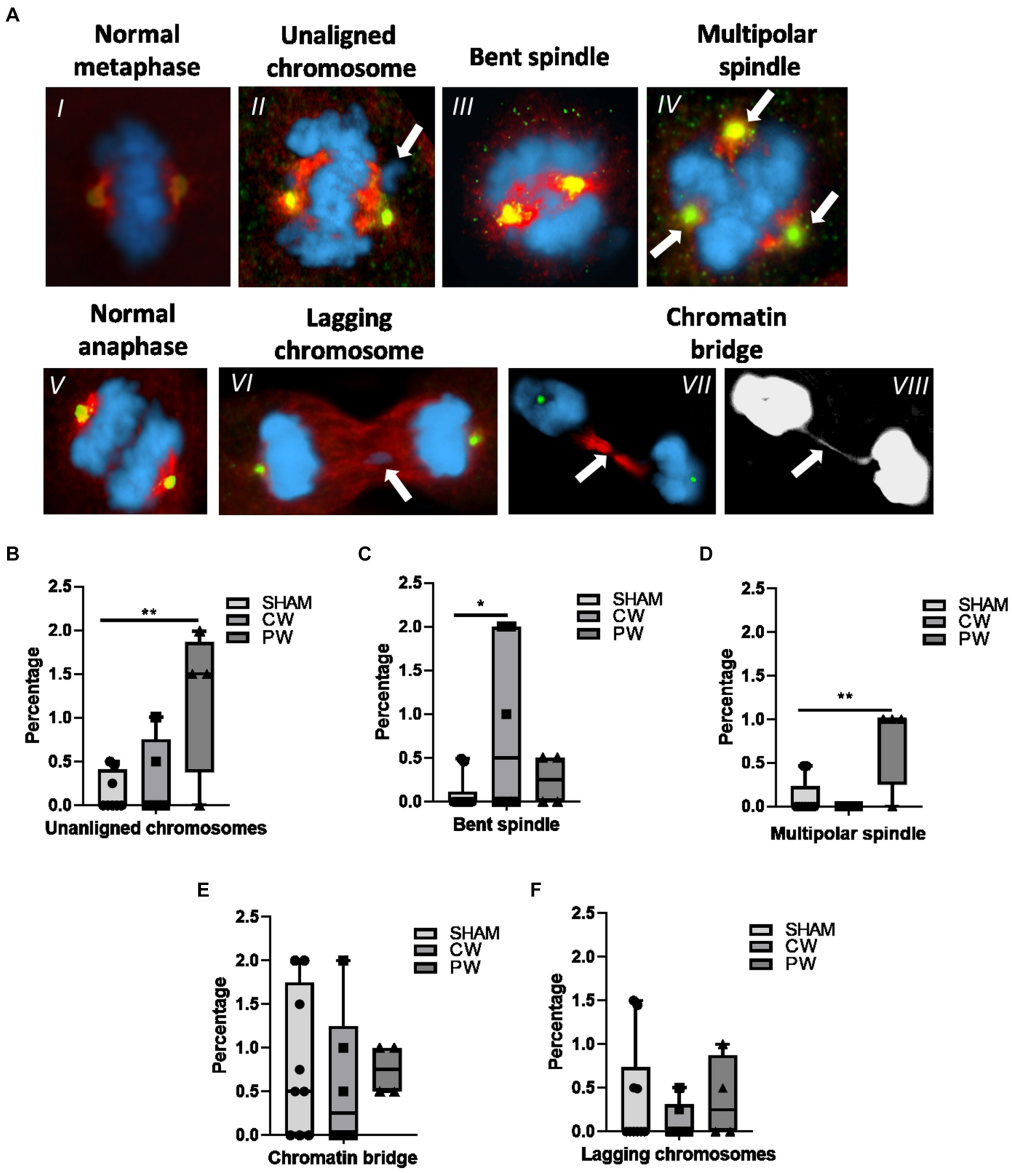
Immunofluorescence staining of γ-Tubulin (green) and α-Tubulin (red) in CW, PW 1.6 GHz RF exposed and sham HDF. **(A)** Representative images of a normal metaphase (I), a metaphase displaying an unaligned chromosome (II) and a bent spindle (III). Representative image of a multipolar spindle (IV) (three centrosomes as indicated by green signals). Representative images of a normal anaphase (V), an anaphase containing a lagging chromosome (VI), and an anaphase displaying a chromatin bridge (VII) in merge and gray scale (VIII). Percentage of unaligned chromosomes **(B)** bent spindles **(C)**, multipolar spindles **(D)**, chromatin bridges **(E)** and lagging chromosomes **(F)** in sham and 1.6 GHz CW and PW RF-exposed HDF cells. At least three independent experiments were conducted.

Potential mitotic spindle organization perturbation following RF-EMF exposure in HDF was investigated using γ-Tubulin and α-Tubulin immunofluorescence (Figure 6A). CW exposure increased the percentage of bent spindles compared to sham cells (Figure 6C). In contrast, CW did not induce unaligned or lagging chromosomes and chromatin bridges. Regarding PW exposure, it is interesting to notice that, although variations in unaligned chromosomes, bent spindles and multipolar spindles were observed, significant variations were found only for unaligned chromosomes and multipolar spindle (Figures 6B,D). In particular, multipolar spindle showed the highest induction after PW exposure, moving from a percentage of 0.10 in the sham cells to a percentage of 0.75 in PW-exposed samples (Figures 6E,F).

### 3.5 Cytokinesis blocked micronucleus assay and CREST staining

**Figure 7:**
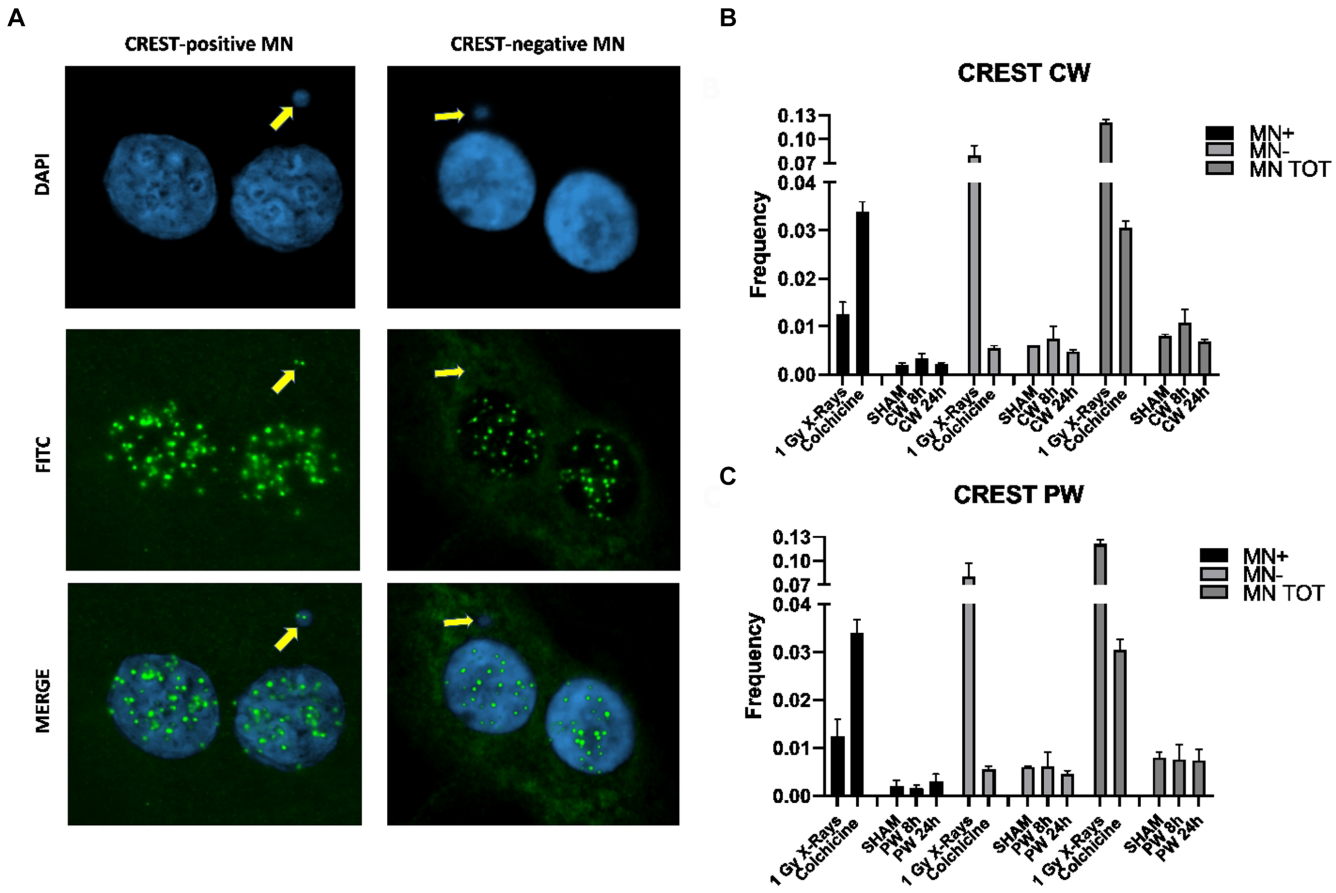
CBMN assay and CREST staining in HDF exposed to 1.6 GHz CW,PW RF. **(A)** Representative images of BN cells with MN CREST-negative (MN-) and MN CREST-positive (MN+) indicated by yellow arrows. **(B)** Bar plots of micronuclei frequencies represent the percentages of CREST-positive, −negative and total MN in HDF sham cells and CW exposure cells. Bars indicate the standard error. **(C)** Bar plots showing the frequencies of CREST-positive, −negative and total MN in HDF control cells exposed to PW. Bars indicate the standard error. As positive control for CREST-negative MN and CREST-positive MN, cells were either irradiated using 1Gy X-rays or treated with 10 ng/mL colchicine. At least three independent experiments were performed.

In order to assess the impact of RF-EMF on chromosome damage, Cytokinesis Blocked Micronucleus (CBMN) assay in combination with CREST immunofluorescence was performed on HDF (Figure 7A). As specified in the materials and methods section, two different protocols were used, the classical protocol (24 h of Cytochalasin-B) and a modified protocol used to restrict as much as possible the analysis on cells exposed to RF during M-phase (8 h of Cytochalasin-B). Using both protocols, it was not observed any variation in total MN neither in CW nor in PW exposed samples (Figures 7B,C).

### 3.6 Chromosome segregation analysis

**Figure 8:**
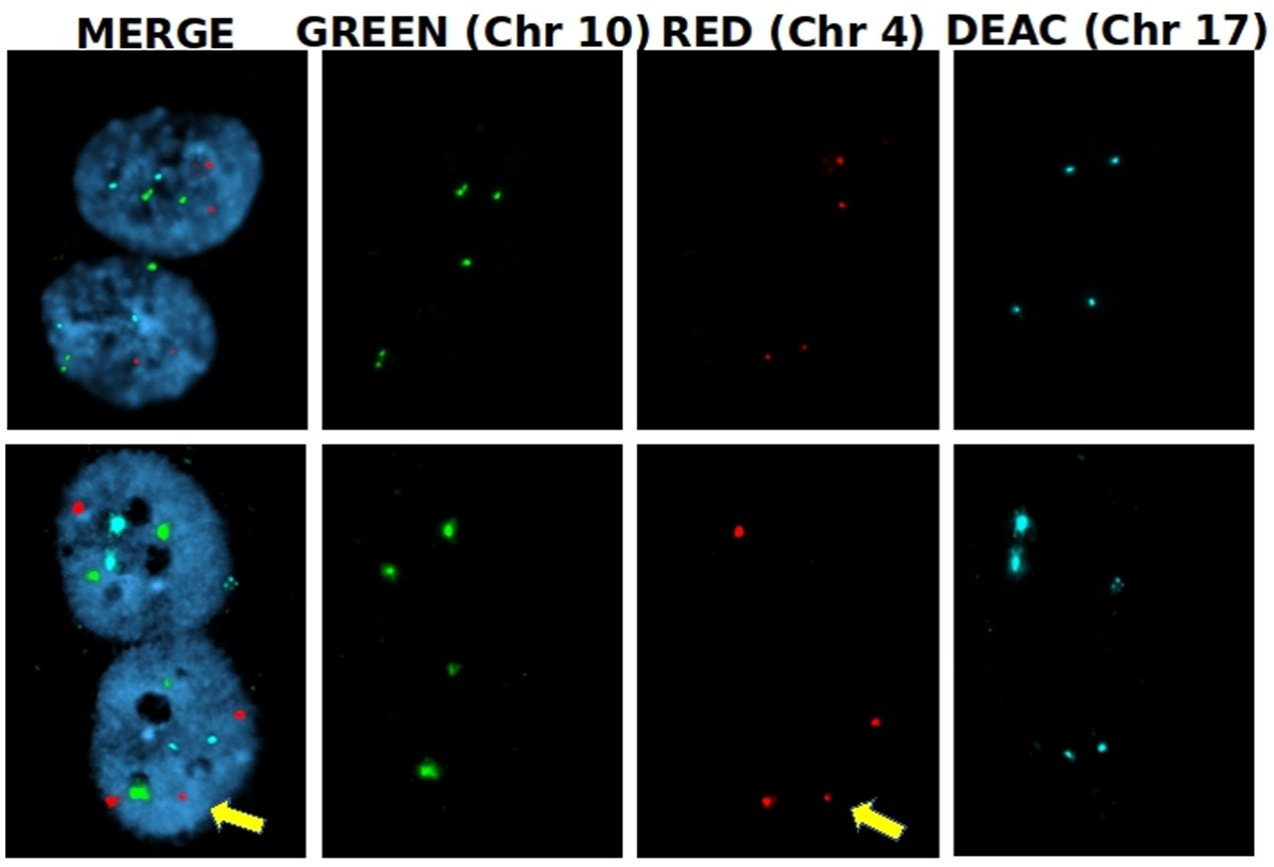
Nondisjunction frequency/percentage in HDF sham and exposed to 1.6 GHz CW RF Representative images of two BN cells displaying a normal chromosome distribution of the three homologous chromosomes (upper row) and an unequal distribution of chromosome 4 (3 + 1) (yellow arrow).

To assess whether RF-EMF induced aneuploidy in HDF, frequency of chromosomes non-disjunction (ND) events was assayed in three different homologous pairs (chromosomes 4, 10, and 17) in BN cells (Figure 8). In both CW and PW exposed samples it was not observed any significant induction of ND events compared to control samples (neither at 8 h nor at 24 h) (Table 2). Despite the lack of significant differences in the frequency of total mis-segregation events in CW and PW exposed samples, it is interesting to notice that ND events have been observed in exposed samples with percentages comprised between 0.07 and 0.14, whereas no ND events were observed in sham samples.

**Table 2:** Non-disjunction frequencies in sham and RF exposed cells.

			Chromosome 4					Chromosome 10					Chromosome 17					4 + 10 + 17
1.6 GHz	Sample	BN	2 + 2	3 + 1	ND%	SD	N	2 + 2	3 + 1	ND%	SD	N	2 + 2	3 + 1	ND%	SD	N	ND%
CW	Sham	5,500	5,500	0	0.00	0.00	6	5,500	0	0.00	0.00	6	5,500	0	0.00	0.00	6	0.00
	8 h	1,505	1,505	0	0.00	0.00	3	1,505	0	0.00	0.00	3	1,504	1	0.07	0.58	3	0.07
	24 h	4,000	3,997	3	0.07	1.00	3	4,000	0	0.00	0.00	3	4,000	0	0.00	0.00	3	0.07
PW	Sham	3,564	3,564	0	0.00	0.00	8	3,564	0	0.00	0.00	8	3,564	0	0.00	0.00	8	0.00
	8 h	2,101	2,098	3	0.14	0.55	5	2,101	0	0.00	0.00	5	2,101	0	0.00	0.00	5	0.01
	24 h	1,531	1,531	0	0.00	0.00	3	1,530	1	0.06	0.58	3	1,531	0	0.00	0.00	3	0.06

Binucleated cells (BN), non-disjunction (ND), standard deviation (SD), and experimental replicates (N).

### 3.7 Analysis of γ-H2AX/53BP1 foci

**Figure 9:**
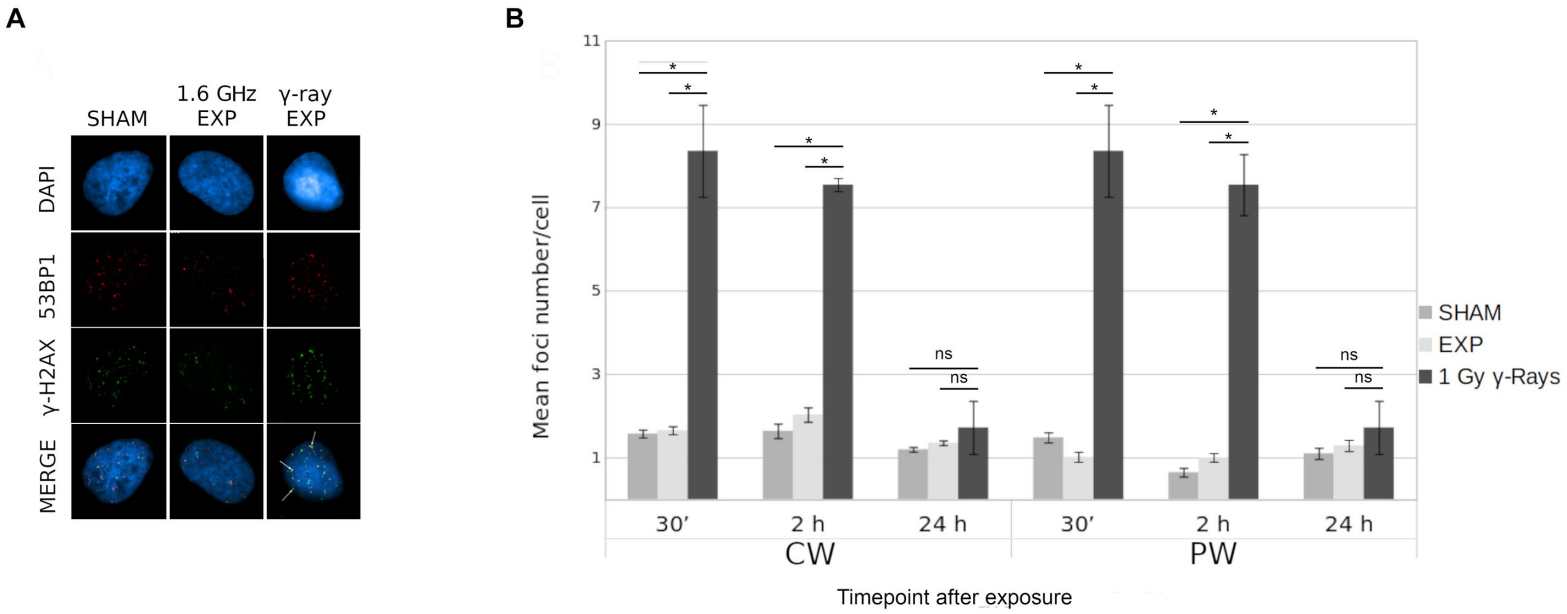
γ-H2AX/53BP1 colocalization foci in in CW, PW 1.6 GHz RF exposed and sham HDF. **(A)** Representative images of γ-H2AX/53BP1 foci acquisition: DAPI (blue), γ-H2AX foci (green), 53BP1 foci (red) and co-localized foci (yellow) in sham cell, 1.6 GHz RF exposed cell and γ-ray exposed cells. Colocalized foci are indicated by white arrows. **(B)** Bar plot of mean H2AX/53BP1 foci/cell in 1.6 GHz CW, PW and in γ-rays exposed and sham cells at 30 min, 2 h, and 24 h after exposure. Bars indicate the standard error. As positive control cells were irradiated at 1 Gy γ-ray. Three independent experiments were performed. (*p ≤ 0.05; ns, *p* > 0.05).

The potential induction of DSBs by RF-EMF in HDF was evaluated by γ-H2AX/53BP1 assay. The effects of both 1.6 GHz CW and PW RF exposure on HDF, were evaluated at 30 min, 2 h and 24 h after RF exposure for each type of signal. Moreover, a γH2AX/53BP1 γ-ray positive control was included. Figure 9A shows representative images of γ-H2AX foci, 53BP1 and co-localized γ-H2AX/53BP1 foci in sham cell, 1.6 GHz RF exposed cell and γ-ray irradiated cell. Results, representative of three experimental replicates, show no significant differences in the mean co-localized γ-H2AX/53BP1 foci between 1.6 GHz RF exposed and sham samples (exposed 2 h vs. sham 2 h; exposed 24 h vs. sham 24 h) for both types of signals (CW or PW) (Figure 9B). Additional data are reported in Supplementary Table S1.

## 4 Discussion

The rapid development of communication technologies based on RF led the scientific community to investigate the biological impact of this non-ionizing radiation, particularly non-thermal effects. However, the results of the current knowledge are controversial, probably due to a lack of standardization in methodologies, homogeneity in cell/animal models and in study design.

This *in vitro* study aims to contribute to a better understanding of the potential non-thermal biological effects of RF exposure in HDF. The frequency of 1.6 GHz RF and a SAR value of 0.4 W/kg (whole body occupational exposure limit of the current European regulation) (4) were employed.

Moreover, different signal modulations, either CW or PW, were used to investigate the different biological impact associated with signal modulation.

To evaluate genotoxic effects, γ-H2AX/53BP1 foci analysis for DSBs, CREST-stained MN and mis-segregation assays for chromosome anomalies were performed. Cellular alterations were investigated by examining cell cycle, HSP and pro-survival proteins expression, ultrastructure and mitotic spindle perturbations potentially induced by RF-EMF exposure.

After a 2 h exposure, either PW or CW, for each time point examined, we did not find any effect on cell cycle. Also, we did not observe genotoxic effects, neither clastogenic nor aneugenic damage, according to the majority of the investigations performed on several cellular models *in vitro* exposed to different RF-EMF frequencies.

Specifically, no variation in the number of total CREST-stained MN, CREST-positive and CREST-negative MN and of γ-H2AX/53BP1 foci was found in human fibroblasts after exposure to RF-EMF as reported in a previous study (13). The absence of MN increase with both signals suggests that 1.6 GHz RF exposure did not induce any chromosomal alteration as reported by several studies (63–67). Also, no significant difference in γ-H2AX/53BP1 colocalized foci, between sham and exposed cells with both CW or PW signal was observed, indicating no induction of DSBs in agreement with the absence of CREST-negative MN.

This result is in accordance with other studies performed on various cell models indicating no increase of γ-H2AX foci number (51, 68, 69). A study in which γ-H2AX/53BP1 foci induction was evaluated in human dermal fibroblasts after PW and CW 2.54 GHz exposure also did not describe any significant differences between sham and exposed samples for both signals (13).

In addition, although our results did not evidence a significant induction of chromosome mis-segregation, it is worth noting that data were collected analyzing only three out of 23 chromosomes. The difference in non-disjunction between exposed and sham samples could have been more representative if the number of chromosomes analyzed was higher. Using a correction factor of 23/3 (the ratio between the pairs of all human chromosome and the chromosomes analyzed) we could extrapolate a percentage of non-disjunction on the whole genome comprised between 0.5 and 1% for CW and PW exposed samples, suggesting that further experiments will be needed to address the biological relevance of such events.

Mitotic spindle analysis following exposure to PW indicated a significant rise in the frequency of multipolar spindles and unaligned chromosomes, while cells exposed to CW exhibited an increase in bent spindles, as compared to sham cells.

It is well known that unaligned chromosomes may recover their alignment on the equatorial plate or display a delay in cell segregation forming lagging chromosomes (70, 71). However, our data did not evidence the presence of lagging chromosomes suggesting that the latter phenomenon did not occur in our experimental setting. On the other hand, both bent and multipolar spindles have been associated to chromosome mis-segregation (72, 73).

It is worth noting that multipolar spindle development can be caused by different cellular events. For instance, centriole overduplication, fragmentation of the pericentriolar material (PCM) or cytokinesis failure in human cells can lead to the formation of multipolar spindles, accompanied by centrosome amplification during mitosis (74, 75). A number of researchers have hypothesized that also non-ionizing radiation may affect the normal formation of spindle poles (63, 76, 77). Specifically, components of the electric field in radiofrequency could disturb the moment of the electric dipole of tubulin, generating mitotic spindle abnormalities as previously proposed (25). It is worth noting that multipolar spindles may contribute to non-disjunction events, as suggested by Thompson and Compton in 2011 (72). Despite this, it is known that multipolar spindles observed during mitosis frequently evolve into normal bipolar spindles, primarily due to the clustering of centrosomes, facilitating progression through normal mitosis (78). However, the clustering of centrosomes can have a side effect in the mis-segregation, resulting from a stronger attachment of two segregating chromosomes to the same pole of the spindle (merotelic attachment). Equi-merotelic attachment (those with approximately equal numbers of microtubules orientated toward the correct and wrong poles) evolves in lagging chromosome in anaphase that end up as micronuclei. Instead, multi-merotelic attachments (those with many microtubules oriented toward the wrong pole) make a large contribution to mis-segregation without displaying lagging in anaphase (72). This phenomenon may offer a partial explanation for those rare non-disjunction events observed in RF exposed cells, suggesting a mechanism that could correlate the presence of multipolar spindle with non-disjunction events and, as a consequence, the absence of lagging chromosomes and CREST-positive micronuclei.

Besides on mitotic spindle, significant effects were found on cell ultrastructure and protein expression after 2 h CW or PW exposure. Morphological observation employing transmission electron microscope showed an increased deposition of filaments below the plasma membrane, similar to what was previously reported in the same cell line for other EMF frequencies (25, 79). The expression of actin, as detected in western blotting assays, was similar in 1.6 GHz RF exposed and sham cells, thus suggesting that the increase of deposition of filaments below the plasma membrane might be due to a different polymerization of filaments rather than to an increase of the synthesis of filamentous proteins. Several double membrane-surrounded lysosomes were observed 24 h after PW exposure, suggesting the formation of autophagosomes and activation of autophagy as pro-survival cell signaling. Activation of autophagy mediated by ROS generation was previously observed in mouse spermatocyte-derived cells (GC-2) and in spiral ganglion neurons (SGN) exposed to 1800 MHz (GSM talk signal) for 24 h, as showed by the formation of autophagosomes, increased levels of LC3-II, Beclin1 and decreased levels of the protein p62 (50, 51). AKT has a key role in autophagy induction. Indeed, in response to the increase in ROS levels under nutrient-rich conditions, AKT inhibits the initiation of autophagy (80). We observed a decrease in activation by phosphorylation of the AKT kinase after exposure to 1.6 GHz RF-EMF.

During stress response, the highly conserved proteins called heat shock or stress proteins (HSPs, categorized according to their size in families HSP-70, HSP-27, HSP-60, HSP-90, and HSP-100), are rapidly produced to protect cells from stressful conditions (heat shock, nutrient deprivation, ultraviolet exposure, and others), mainly through their ability to prevent misfolding of other proteins and to accelerate their refolding and renaturation (81). No uniform data on the expression of HSPs after RF exposure are reported in the literature. This is probably due to the cell type employed, exposure modality or endpoint chosen for the experiments in each study. Gerner et al. reported that mitochondrial HSP-60, HSP-70, and HSP-90 were found to increase, among several more proteins, in cultured human fibroblasts exposed to 1800 MHz (exposure time of 8 h with intermittence pattern 5 min. ON /10 min OFF) (82). Conversely, Sanchez et al. reported that 900 MHz RF had no effects on HSP-27 or HSP-70 expression after 48 h of exposure of human primary dermal fibroblasts (83). Similarly, IMR-90 human fibroblasts exposure to 2.1425 GHz CW for up to 28 h did not vary the expression of HSP-27 and HSP-70 (84). In human peripheral blood mononuclear cell, exposed to 1.8 GHz RF-EMF for up to 44 h, the HSP-70 level, as analyzed by flow cytometry, was not modified (85). In our experiment, we found an increased expression of only HSP-60 and HSP-25 in HDF after 2 and 24 h of 1.6 GHz PW RF exposure, suggesting an activation of these proteins. In contrast, we observed a decreased expression of HSPs 24 h after a CW RF exposure.

In this controversial scientific scenario, our results are in agreement with most *in vitro* findings on different cell models about the lack of genotoxic damage induced by RF, either clastogenic or aneugenic. Despite this, we find alterations of the mitotic spindle, with a significant increase in multipolar spindles following PW exposure and we also observed a tendency of RF to induce non-disjunction events with both signals. However, our study reveals an increase in these spindle abnormalities without a concomitant increase in MN-positive CREST, suggesting no aneuploidy effect probably due to spindle abnormalities reversion, as proposed by other authors (78). Nonetheless, this apparent discordant result requires further investigations.

The reported observations indeed highlight the complexity of cellular response to RF and emphasize the need for further investigations to clarify the overall biological effects of 1.6 GHz PW and CW RF, given the widespread and constant public RF-EMF exposure, mostly due to mobile communication devices.

## Data availability statement

The raw data supporting the conclusions of this article will be made available by the authors, without undue reservation.

## Ethics statement

Ethical approval was not required for the studies on humans in accordance with the local legislation and institutional requirements because only commercially available established cell lines were used.

## Author contributions

LuM: Data curation, Investigation, Methodology, Writing – original draft, Writing – review & editing. SDS: Conceptualization, Data curation, Methodology, Project administration, Supervision, Writing – original draft, Writing – review & editing. VF: Data curation, Formal analysis, Investigation, Writing – review & editing, Writing – original draft. ElR: Conceptualization, Data curation, Formal analysis, Investigation, Writing – review & editing, Writing – original draft. GA: Investigation, Writing – review & editing, Data curation. CF: Writing – review & editing, Formal analysis. MB: Writing – original draft, Writing – review & editing, Data curation, Formal analysis, Investigation, Validation, Visualization. LC: Writing – review & editing. AS: Conceptualization, Data curation, Funding acquisition, Project administration, Supervision, Writing – original draft, Writing – review & editing. FrB: Writing – review & editing, Conceptualization, Data curation, Formal analysis, Investigation, Methodology, Supervision, Writing – original draft. JM: Writing – review & editing, Conceptualization, Data curation, Formal analysis, Investigation. FeB: Writing – review & editing, Formal analysis, Investigation. ErR: Writing – review & editing, Formal analysis, Investigation. DN: Writing – review & editing, Formal analysis, Investigation. LaM: Conceptualization, Data curation, Formal analysis, Investigation, Methodology, Supervision, Validation, Visualization, Writing – original draft, Writing – review & editing. RB: Conceptualization, Formal analysis, Funding acquisition, Methodology, Project administration, Resources, Supervision, Writing – review & editing. FL: Conceptualization, Data curation, Funding acquisition, Methodology, Project administration, Resources, Supervision, Writing – original draft, Writing – review & editing.
