# Supplementary material for: Study of genotoxic and cytotoxic effects induced in human fibroblasts by exposure to pulsed and continuous 1.6 GHz radiofrequency
Source: Front Public Health. 2024 Jul 31;12:1419525. doi: 10.3389/fpubh.2024.1419525 (PMC11323689; doi:10.3389/fpubh.2024.1419525)
Supplement: Supplementary file 1 [file Data_Sheet_1.docx]

Supplementary Material

Study of genotoxic and cytotoxic effects induced in human fibroblasts by Exposure to pulsed and continuous 1.6 GHz radiofrequency

FMassaro, Luca* ^1,2^; De Sanctis, Stefania ^1^; Franchini, Valeria ^1^; Regalbuto, Elisa ^1^; Alfano, Gaetano ^1^; Focaccetti, Chiara ^2^; Benvenuto, Monica ^2^; Cifaldi, Loredana ^2^; Sgura, Antonella ^3^; Berardinelli, Francesco ^3^; Marinaccio, Jessica ^3^; Barbato Federica ^3^; Rossi, Erica ^3^; Nardozi, Daniela ^4^; Masuelli, Laura ^4^; Bei, Roberto^2^; Lista, Florigio^1^

^1^Defence Institute for Biomedical Sciences, Rome, Italy

^2^University of Rome “Tor Vergata”- Department of Clinical Sciences and Translational Medicine, Rome, Italy;

^3^University of Rome “Roma Tre”- Department of Science, Rome, Italy;

^4^University of Rome “La Sapienza”- Department of Experimental Medicine, Rome, Italy

*** Correspondence:**luca.massaro@alumni.uniroma2.eu

# Supplementary Data

**1.1 Exposure system and characterization**

To perform the Exposure of cells, an Exposure system was designed and developed, capable of emitting a continuous (CW) or a pulsed wave (PW) at a frequency of 1.6 GHz.

The chain necessary for the generation of the signal feeding the TEM cell terminated with a 50 Ω load, that is the core of the Exposure system inside of which the EMF is generated, and the Petri dishes were placed, is shown in Fig. A.1 where the chain is represented as a block diagram. As shown in Fig. A.1 the TEM cell (Open TEM cell *TEM3000*, Montena) is placed inside an incubator (*Galaxy S CO2 Incubator*, model n° 170-200) able to real-time control the humidity and the temperature of the environment during the Experiments for the cell viability. The TEM cell has overall dimensions of 40 x 18 x 6 cm^3^ and four Petri dishes of 35 mm of diameter and height of 10 mm were placed under the septum of the TEM where it is proved to be the region with the best Exposure uniformity. The inner-walls of the incubator were covered by radio-absorbing panels (*Flat Single Layer Absorbed P25*, Siepel) in order to avoid the contribution of possible reflections due to the metallic walls of the incubator.

The TEM cell is fed by an electric signal generated by the RF signal generator (*Keysight N9310A*, Keysight Technologies), and amplified by the power amplifier (*FLG-10CA*, Frankonia Group); all these three blocks are connected through coaxial cables.

In order to monitor real-time the amount of net input power feeding the TEM-cell, the incident power and the reflected one were monitored during the Experiments. This was done by connecting the power amplifier to a bidirectional coupler (*RFDDC5M06G40*, RF-LAMBDA) connected itself to two power meters (*PMU6003*, Teseq), one used to measure the incident power and one used to measure the reflected power, and, from their difference, the net input power to the TEM. Both the power meters were connected via USB cable to a dedicated computer in order to visualize, real-time, the values of the incident and reflected power thanks to a software interface provided by the producer. During all the Experiments, the values of both the incident and the reflected power resulted stable, with a mean reflected power always less than the 5% of the incident one.

Furthermore, inside the incubator a temperature control system based on a fan was used to induce a forced air flow and the Exposure system was equipped with a temperature probe (*Fiber Optic Temperature*, FISO, temporal sampling step of 30 s) connected through a fiber optic cable with an external temperature readout (*FOR-1 Single Channel Portable Readout*, Smartec) for monitoring the temperature into the Petri dish. Temperature measurements were carried out in separate Experimental sessions under the same Experimental RF-Exposures (both CW and PW) in order to check that the conditions for the occurrence of thermal effects were not triggered. Independent measures performed under both CW and PW conditions revealed that the peak temperature increase during 2 hours of Exposure was 0.35°C, that is well below the threshold for the onset of thermal effect [1]. Fig. A.2 shows an example of the temperature increase (ΔT (°C)) measured during 2 hours of PW Exposure. As evident from the temperature trend shown in figure A.2 (see light blue line), after 2 hours, the estimated increase of temperature was of 0.2 °C, whereas the greatest peak of ΔT was 0.30° C.In order to assess the level of the uniformity of the Exposure, a dosimetric analysis was carried out by means of a computational approach to evaluate both the uniformity of the EMF produced inside the TEM cell, considering also when it is placed inside the incubator equipped with the absorbing panels, and the Specific Absorption Rate (SAR) distribution inside the biological samples.

With more detail, in the first phase of the numerical characterization of the Exposure system, an unloaded TEM cell was simulated. Numerical simulations were carried out to quantify the electromagnetic field pattern to which the Petri dishes will be Exposed to. This analysis was developed using the commercial software CST Microwave Studio© (Simulia CST Studio Suite) based on the Finite Integration Technique to solve Maxwell’s equations. The TEM cell was modeled according to the shape and dimension of the real one and made of copper (σ = 5.96x10^7^ S/m). A hexahedral mesh was adopted with 20 lines per wavelength, being the best compromise between the accuracy and the computational cost. The TEM cell was fed by a sinusoidal voltage signal with frequency of 1.6 GHz at a tapered section while the other tapered section was assumed to be terminated with a 50 Ω load. The simulations were performed on a workstation Z8 16-Core Processor @3.8 GHz, RAM 512 GB, with a graphical card NVIDIA GeForce RTX5000. In Figure A.3 a transverse section of the electric field generated inside the TEM cell, obtained with the aforementioned conditions, is shown with also the vector representation of the electric field itself. This section was extracted in correspondence of the central slice of the simulated TEM cell. The same analysis was also performed when the TEM cell was placed inside the incubator, the inner walls of which were covered with radio-absorbing panels. The incubator was modeled as an empty cube made of polyimide (σ = 6.62x10^-7^ S/m, ε_r_ = 3.5), the panels were simulated as an inner layer of polyurethane (σ = 1x10^-8^ S/m, ε_r_ = 3.4) and the TEM was placed inside the incubator in its realistic position. The entire system was discretized with a hexahedral mesh and the field distributions were obtained with the same feeding conditions of before. The comparison of the field distributions obtained inside the TEM cell when it is placed inside or not the incubator equipped with the absorbing panels revealed that the presence of these elements does not affect the electric field distribution and its uniformity, particularly in the area under the septum, where the Petri dishes are located.In the second phase of the numerical characterization of the Exposure system, a dosimetric characterization was performed, assessing the Specific Absorption Rate (SAR) distribution inside the biological samples. Simulations were performed using the platform software Sim4Life v.5.2 (ZMT Zurich Med Tech AG, Zurich, Switzerland, [www.zurichmedtech.com](http://www.zurichmedtech.com/)) where the Finite-Difference Time-Domain (FDTD) method is implemented. The Petri dishes were modeled as closed cylinders made of polystyrene (σ =0.00047 S/m, ε_r_= 2.54), filled with 2.7 ml of blood (σ=1.91 S/m, ε_r_=59.738 and density ρ=1049.75 kg/m^3^ [2]) and placed inside the TEM cell. The computational domain was discretized with an adaptive non-uniform grid with a sub-wavelength resolution of around 20 mesh cells per wavelength in order to correctly discretize all the computational domain to guarantee the compliance with the constraint imposed by the FDTD method for its stability. The computational domain was truncated by assuming 8 layers of perfectly matched layer (PML) material and 10 cells of free space were added around the computational domain at the domain boundaries. The feed was a sinusoidal signal with a frequency f = 1.6 GHz and 1W of input power. The simulations were performed on a workstation Z8 16-Core Processor @3.8 GHz, RAM 512 GB, with a graphical card NVIDIA GeForce RTX5000 equipped with Sim4Life GPU accelerator aXware.

The performed simulations revealed that the uniformity of the electric field under the septum is almost 70% with a SAR nonuniformity degree acceptable according to the literature data [3].

# Supplementary Figures and Tables

## Supplementary Figures

Supplementary Figure 1: Block diagram of the Exposure system used for the Experiments.

Supplementary Figure 2: Temperature increase as a function of time. The measurement was performed inside the biological sample during 2 hours of Exposure to a PW at 1.6 GHz at 0.4 W/kg.

Supplementary Figure 3: E-field pattern in a transverse section with also its vector representation

**Supplementary Figure** 4: Expression of HSPs proteins performed 24 hours after CW Exposure. Western blotting analysis was performed on three different HDF Sham samples (SHAM OFF: Petri dishes with HDF placed in the incubator when the RF-EMF is **s**witched OFF; SHAM TEM OFF: Petri dishes with HDF placed in the TEM cell when the RF-EMF is switched OFF; SHAM: Petri dishes with HDF placed in the incubator but outside the TEM cell when the RF-EMF isswitched ON) in addition to the Exposed sample (EXP: Petri dishes with HDF placed in the TEM cell when the RF-EMF is switched ON). Actin was used as an internal control. Densitometric ratios are reported. Mean densitometric values of SHAM sample bands (SHAM) were set to 1.0. Data are expressed as the mean ± SD of two independent experiments.

**Supplementary Figure** 5: Uncropped Western blots corresponding to Figures 4, 5 and Suppl. Fig. 4. Note that the membranes were cut before probing.

**2.2 Supplementary tables**

**Table 1** *In* *the table are reported, the average γ-H2AX/53BP1 foci number for time point (Sham and Exposed) and average total foci count. The results are presented in the format of mean±SE*

| ‍ | Timepoint after exposure | | | | | | | | | | | | | | |
| --- | --- | --- | --- | --- | --- | --- | --- | --- | --- | --- | --- | --- | --- | --- | --- |
|  | 30 min | | | | | 2 h | | | | | 24 h | | | | |
|  | Sham/control | Total | Exp | Total | p^a^ | Sham/ control | Total | Exp | Total | p^a^ | Sham/ control | Total | Exp | Total | p^a^ |
| CW | 1.57±0.09 | 381 | 1.66±0.17 | 403 | 0.801 | 1.64±0.25 | 458 | 2.03±0.29 | 407 | 0.449 | 1.19±0.08 | 253 | 1.35±0.11 | 267 | 0.404 |
| PW | 1.48 ±0.23 | 294 | 1.01 ±0.09 | 202 | 0.198 | 0.73 ±0.1 | 130 | 1.08 ±0.26 | 188 | 0.446 | 0.96 ±0.26 | 205 | 1.19 ±0.23 | 244 | 0.729 |
| 1 Gy γ-Rays | 2.45±0.36 | 469 | 8.35±1.01 | 1566 | 0.043 | 2.325±0.46 | 383 | 7.54±0.73 | 1645 | 0.0231 | 1.55±0.57 | 279 | 1.72±0.63 | 336 | 0.91 |

*a Significance of the effects comparing Exposed vs. Sham HDF cells was calculated employing the 2-tailed paired Student’s t-Test.*

**Bibliography**

1. M. Simkó, D. Remondini, O. Zeni, O, M.R. Scarfi. Quality Matters: Systematic Analysis of Endpoints Related to “Cellular Life” in Vitro Data of Radiofrequency Electromagnetic Field Exposure. Int. J. Environ. Res. Public Health 2016, 13, 701.
2. S. Gabriel, R. W. Lau, and C. Gabriel. "The dielectric properties of biological tissues: III. Parametric models for the dielectric spectrum of tissues," Physics in medicine & biology, vol. 41, p. 2271, 1996.
3. N. Kuster and F. Schönborn, “Recommended minimal requirements and development guidelines for Exposure setups of bio-Experiment addressing the health risk concern of wireless communication,” Bioelectromagnetics, vol. 21, pp. 508–514, 2000
