# Supplementary figures and images for: Study of genotoxic and cytotoxic effects induced in human fibroblasts by exposure to pulsed and continuous 1.6 GHz radiofrequency
Source: Front Public Health. 2024 Jul 31;12:1419525. doi: 10.3389/fpubh.2024.1419525 (PMC11323689; doi:10.3389/fpubh.2024.1419525)

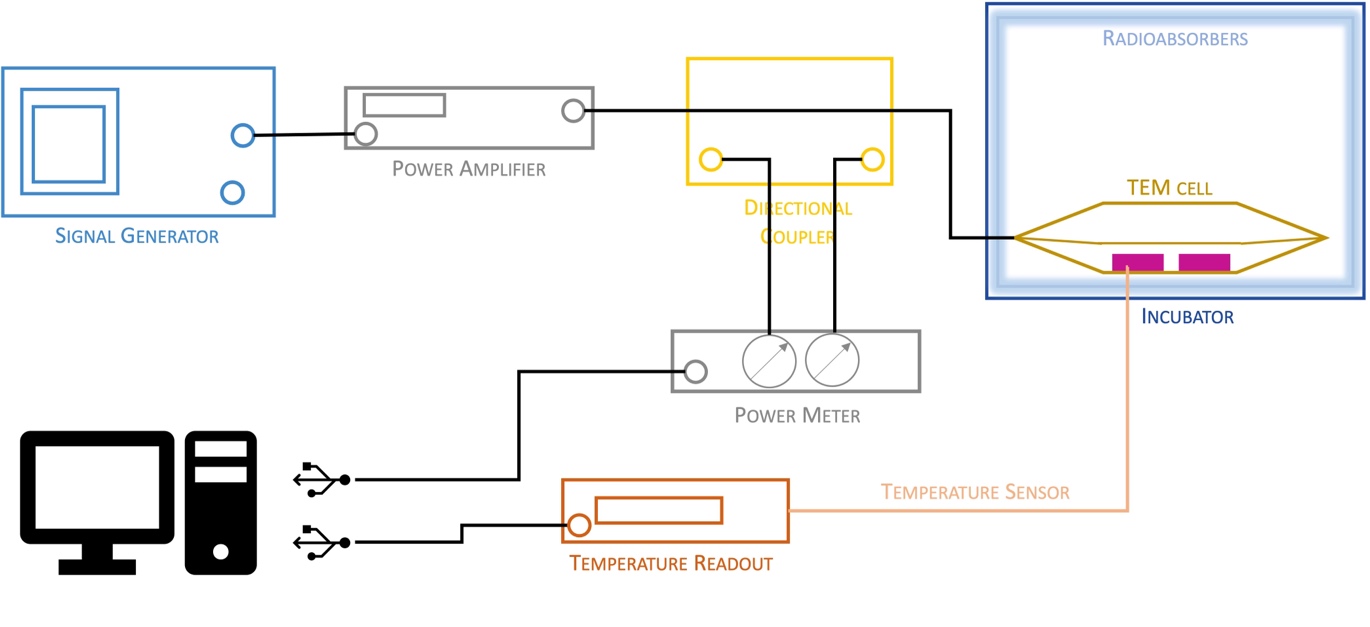

Supplement: Supplementary file 2 [file Image_1.JPEG]

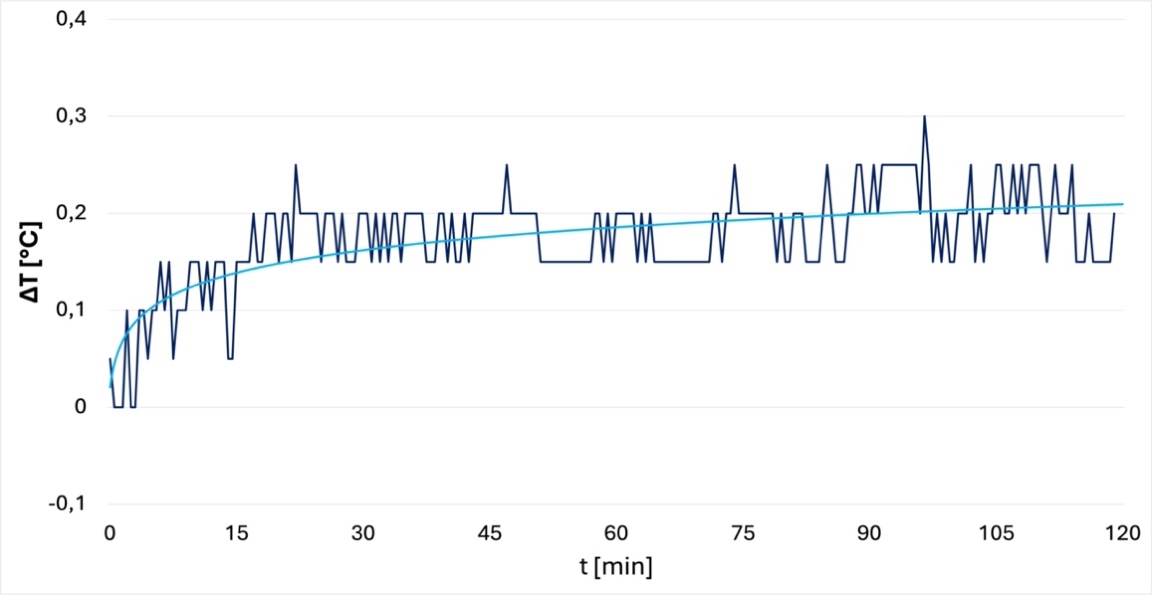

Supplement: Supplementary file 3 [file Image_2.JPEG]

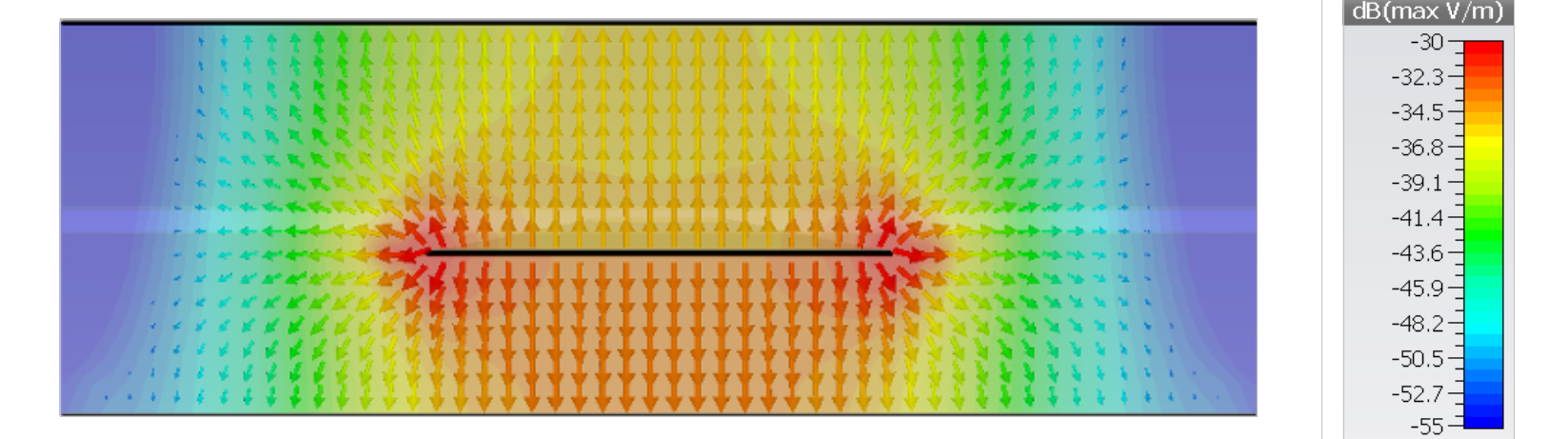

Supplement: Supplementary file 4 [file Image_3.PNG]

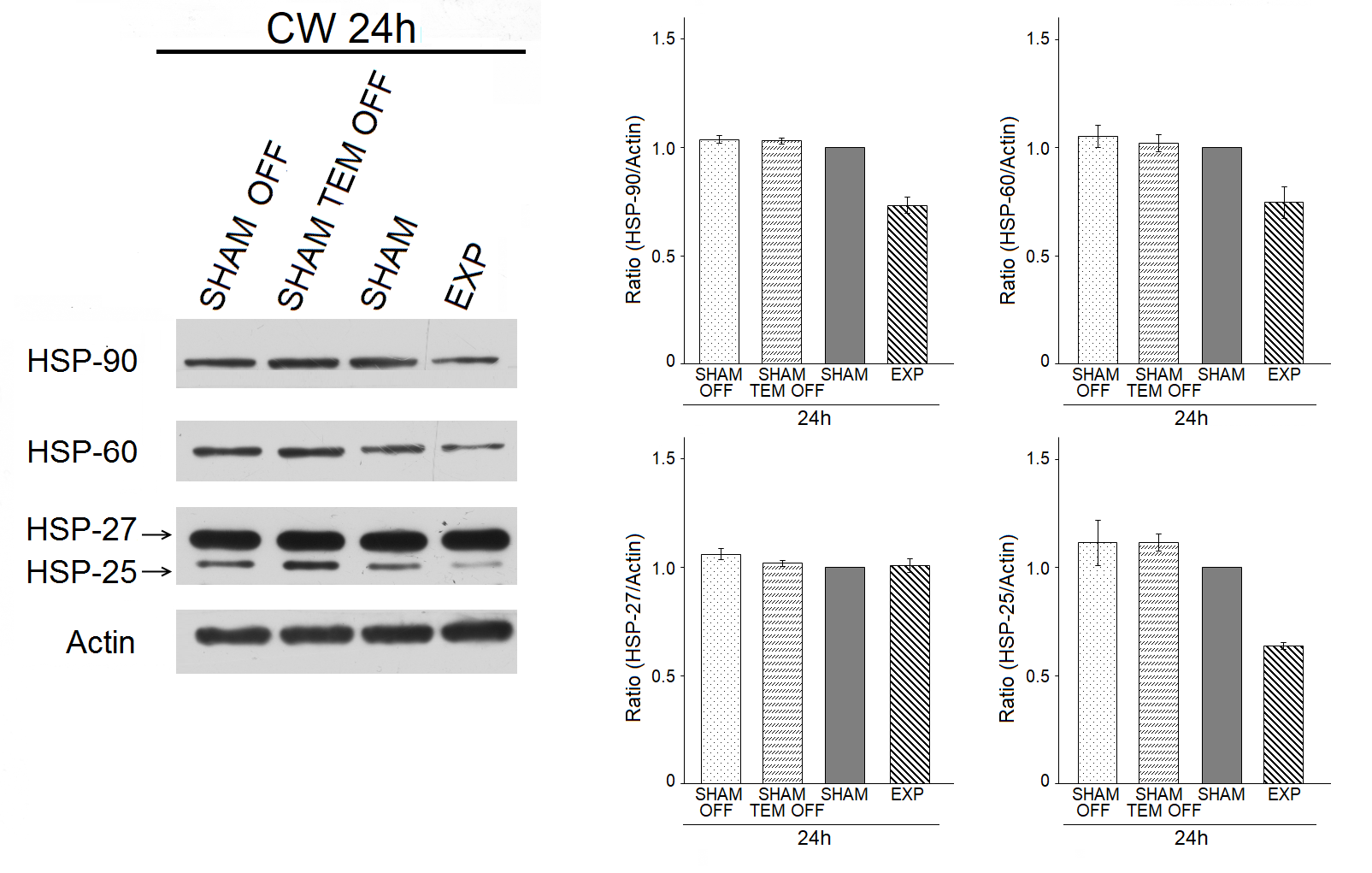

Supplement: Supplementary file 5 [file Image_4.TIF]

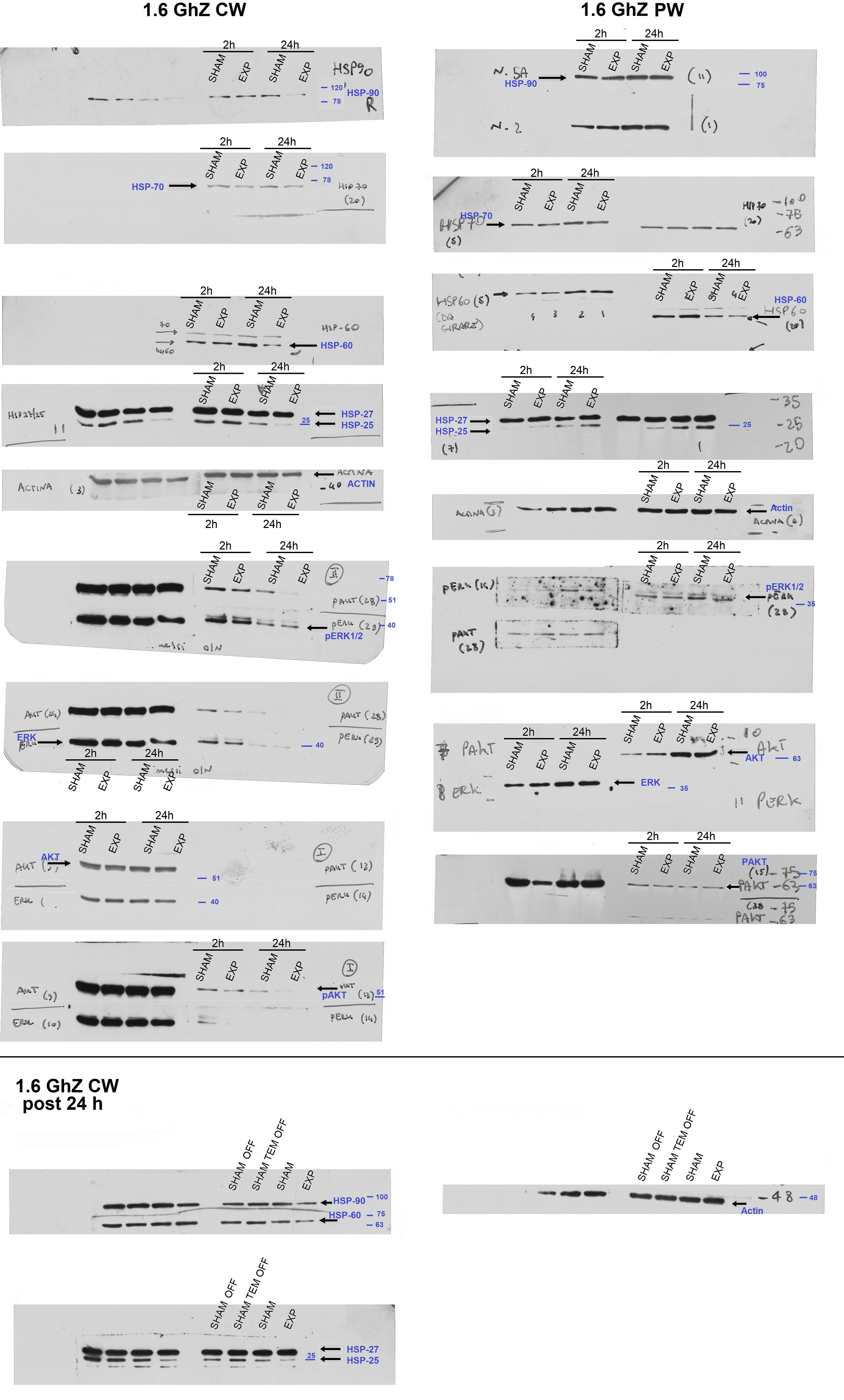

Supplement: Supplementary file 6 [file Image_5.TIF]
